# ACI Alliance – The American Cochlear Implant Alliance Foundation

**DOI:** 10.1179/1467010013Z.00000000082

**Published:** 2013-03

**Authors:** John K Niparko, Teresa Zwolan

**Affiliations:** 1 Keck School of Medicine of USC, Los Angeles, USA; 2 University of Michigan Cochlear Implant Program, Ann Arbor, USA

Cochlear implants enable children and adults who are deaf to hear the ever present sounds of speech, music, and the environment in their daily lives. Without intervention, adults and children with severe-to-profound hearing loss suffer great consequences, such as delays in the development of spoken language, reduced academic performance, and reduced occupational success. The successful utilization of cochlear implantation has dramatically changed the long-term effects of deafness. The reduction in disability and the societal benefits of this technology make it one of the most cost-effective treatments in all of modern medicine.

There are numerous professionals who play instrumental roles in the care of cochlear implant recipients, including surgeons, audiologists, speech-language pathologists, and educators. These professionals work as a team to maximize outcomes obtained by patients from this technology. Additionally, cochlear implant manufacturers assist patients and their families with technological support for external equipment, user networks, and by providing on-line rehabilitation and training tools.

Of the estimated population of candidates in the USA who could benefit from a cochlear implant, only a small percentage has received one. Barriers to cochlear implantation can be traced to low awareness of this proven, time-tested solution among both the general public and the medical community. Further, there are no universally sanctioned medical/clinical guidelines for best practices in cochlear implantation and audiology after care – the data-based, best practices which lead to consistent clinical outcomes. Low reimbursement through public and private payers along with recent reductions in Medicaid payment has only increased the financial burden. While there are societies that represent the needs of the diverse populations of those with hearing loss, there is no independent organization with a dedicated, accountable, and organized approach addressing access to cochlear implantation on behalf of patients.

In 2010, a group of surgeons, audiologists, speech-language pathologists, and representatives of cochlear implant manufacturers in the USA met to discuss the initiation of an organization that would address the many issues cited above. This group determined that an inclusive and collaborative organization was needed to eliminate barriers to cochlear implantation. It was determined that this organization would drive change by improving access to, and increasing awareness about cochlear implants through a concerted effort among health care professionals, patients and their families, corporations, clinics, and hospitals.

In October 2011, the American Cochlear Implant Alliance Foundation, ‘ACI Alliance’ was established as a Delaware not-for-profit 501(c) (3) corporation. Its tax exempt status application has been filed and is under review. A Board of Directors has been elected and consists of some of the country's top professionals who work in the area of hearing loss and cochlear implantation as well as those personally touched with hearing loss. To ensure complete autonomy none of the manufacturers are involved in the organization or have voting eligibility, but each did provide seed monies for the establishment of the Alliance. Over the course of several months its founding members honed the organization's mission and its commitment as follows:

The mission of the ACI Alliance is to advance access to the gift of hearing provided by cochlear implantation through research, advocacy and awareness.

It is committed to eliminating barriers to cochlear implantation by sponsoring research, driving heightened awareness, and advocating for improved access to cochlear implants for patients of all ages across the USA.

To do this the ACI Alliance aims to:

1. Conduct awareness campaigns among the general public, patients, and medical community.
2. Develop and/or sponsor clinical trials and research documenting benefits and demonstrating links to overall health and wellness.
3. Educate health care plan executives and government officials about CI technology, its economic and social benefits, and the relative value associated with coverage.
4. Organize collaborative efforts to foster new research and encourage best clinical practices for standardized outcomes.

As a newly established not-for-profit ACI Alliance will be advocating for the patient population in hopes of benefiting the public at large. Its business model is built upon the premise of a lean and efficient ‘virtual’ organization and to not have overhead costs of bricks and mortar so that it may be agile and invest as much support as possible to its mission. During its first year of establishment the foundation has been laid with the election of a board of directors, adoption of governance documents, regulatory filings, budget development, strategic planning, launch of an initial website, and membership drive.

ACI Alliance has also been involved in several instrumental projects over the past few months. A small group of members developed and submitted a proposal to the Center for Medicare and Medicaid Services (CMS) to evaluate revised and expanded indications for cochlear implant candidacy for the adult CMS population. The proposal includes development of a nationwide registry for collection of CI recipient data that can assist with topics such as validation of indications, efficacy results, patient outcomes in terms of quality of life, speech perception and performance over time, incidence and follow-up of issues, explantation and reimplantation, and equipment failures, to name a few. CMS has indicated that the Alliance's original proposal has been judged as credible and that it has met the requirement for response issued by the CMS in the spring of 2011. The feedback from CMS included a 19-point list of clarifications, and a response from ACI Alliance is currently being developed.

For more information about ACI Alliance, its Board of Directors and how to become a member, and to contact the new Executive Director, Donna Sorkin, visit www.acialliance.org.

